# Host-parasite interactions in non-native invasive species are dependent on the levels of standing genetic variation at the immune locus

**DOI:** 10.1186/s12862-020-01610-x

**Published:** 2020-04-16

**Authors:** Aleksandra Biedrzycka, Marcin Popiołek, Andrzej Zalewski

**Affiliations:** 1 Institute of Nature Conservation, Polish Academy of Sciences, Al. Adama Mickiewicza 33, 31-120 Kraków, Poland; 2 Department of Parasitology, Institute of Genetics and Microbiology, University of Wrocław, Przybyszewskiego 63/67, 51-148 Wroclaw, Poland; 3 Mammal Research Institute, Polish Academy of Sciences, ul. Stoczek 1, 17-230 Białowieża, Poland

**Keywords:** Raccoon, MHC-DRB, Intestinal parasites, Digenea, Cestoda, Invasive populations, Immune genetic diversity, Standing genetic variation, Genetic drift

## Abstract

**Background:**

Parasites may mediate the success of biological invasions through their effect on host fitness and thus, on host population growth and stability. However, a release from the pressure of parasites is strongly related to the genetic differentiation of the host. In invasive host populations, the number of available genetic variants, allowing them to ‘fight’ the infection, are likely to be influenced by founder events and genetic drift. The level standing genetic variation of invasive populations may be crucial in successfully adapting to new environments and resisting diseases. We studied invasive populations of raccoon that experienced a random reduction in genetic diversity during the establishment and evaluated the relationship between host immune genetic diversity and intestinal parasites infection.

**Results:**

We distinguished two different genetic clusters that are characterized by different sets of functionally relevant MHC-DRB alleles. Both clusters were characterized by considerably different allele-parasite associations and different levels of parasite infection. The specific resistance MHC-DRB alleles explained the lower prevalence of Digenea parasites. An increased infection intensity was related to the presence of two MHC-DRB alleles. One of these alleles significantly decreased in frequency over time, causing a decrease of Digenea abundance in raccoons in consecutive years.

**Conclusions:**

Our findings suggest that intestinal parasites can exert selective pressure on an invasive host with lowered levels of immune genetic diversity and contribute to promoting local adaptation over time. The random genetic drift that created the two different genetic clusters in the invasive raccoon range imposed completely different MHC-parasite associations, strongly associated with the infection status of populations. Our findings underline the role of standing genetic variation in shaping host-parasite relationships and provide empirical support that functional genetic variation may be, at least partly, responsible for differences in the success of invasive populations.

## Background

Parasites may mediate the success of biological invasions through their effect on host fitness and thus on host population growth and stability [1–3]. According to the enemy release hypothesis, newly established populations of non-native species harbour fewer enemies (pathogens and parasites) in the introduced range compared to the native range, which reduces top-down population regulation and accelerates spatial expansion [4]. However, in an introduced range, non-native populations may come into contact and accumulate novel pathogen species, gaining high infection intensities over a relatively short time (e.g. [5]). This in turn may affect individual condition [6]. The interaction between hosts and parasites is strongly affected by the environment that can, for example, influence parasite abundance or transmission [7–9]. On the other hand, as indicated by a recent meta-analysis of parasite populations invading hosts with different levels of genetic diversity, parasite success is strongly limited by host genetic differentiation [10]. In the case of invasive populations, altered host–parasite relationships or novel host–parasite interactions affect individual and population functioning on the immunological, behavioural, and ecological levels [11, 12]. The immune system of invasive species is predicted to show a high level of plasticity and tends to adapt to sudden changes in antigenic diversity and pressure [11]. The factor that can limit this flexibility is the individual genetic variance underlying the functional diversity of the immune system [13]. The immune response of a host depends on available genetic variants, which on the population level are likely to be influenced by founder events and genetic drift. The levels of invasive populations’ standing genetic variation, created as an effect of population bottlenecks during the introduction of usually low numbers of individuals, maybe crucial for their successful adaptation to new environments and resisting diseases. Over time, during the expansion process of a successful invader, this genetic variation becomes further partitioned among populations as a result of demographic changes and adaptation to local conditions (reviewed in [14]). Although the case of possessing highly diverse immune-related genes may accelerate adaptation via pathogen-mediated selection [15], it strongly depends on the subset of the genetic variants that were introduced as the population was being established. As an effect of genetic drift, allele frequencies may vary considerably between local populations, and different allele-pathogen relationships may arise.

Genes that compose the major histocompatibility complex (MHC) capture an essential fraction of adaptive genetic variation underpinning resistance to pathogens [15, 16] and have proved to be useful in examining the adaptive potential of populations in mammalian species [17–20]. The high polymorphism of MHC has been demonstrated to be maintained by balancing selection driven by pathogens [21–24]. The change in the frequency of resistance or susceptibility alleles is believed to result from parasite-mediated balancing selection. If an allele confers advantages to host, its frequency should increase, whereas an allele is not beneficial, it should become rare over time [25]. Local variation in parasite-mediated selection pressures could induce population differentiation at MHC [26]. Alternatively, balancing selection at the MHC loci may reduce population differentiation. On the other hand, inter-population MHC diversity may be created by genetic drift and the loss of specific alleles, processes that play an important role when an invasive population establishes and spreads. Due to the high functional diversity of receptors, MHC variation is likely to have qualitative effects on the host immune response, because the allelic composition affects the expression of particular receptors that may render a population resistant or susceptible to a specific parasite.

The raccoon, *Procyon lotor,* is a carnivore whose native distribution is in Northern and Central America [27]. Both in native and invasive range, the raccoon is a reservoir of numerous viral (*rabies virus, canine distemper virus*), bacterial (*Leptospira* spp., *Francisella tularensis*), and parasitic (*Baylisascaris procyonis*, *Toxoplasma gondii*) pathogens [28–30]. Raccoons were successfully introduced in Germany in the 1930s and recent genetic analyses suggest that there were at least four, small-scale, independent initial introduction events [31]. Presently, the range of the species in Europe has extended to the west, east, and south of the introduction core area [32]. In its invasive range throughout Europe, a pronounced neutral genetic structure due to multiple founder events and genetic drift was demonstrated [31, 33]. The pattern of population differentiation was, to some extent, mirrored in the immune MHC-DRB locus [34]. Our previous research analysed the population structure displayed by this immune-related locus in raccoon populations located at the invasion core in central Germany up to the eastern invasion edge in western Poland and the southern edge in Czechia (formerly Czech Republic). We identified two distinct population clusters reflected by both neutral microsatellite markers and mitochondrial DNA as well as the MHC-DRB locus diversity, which proved different foundations of the studied invasive populations. Raccoon populations from Poland and Germany form a relatively homogenous cluster. The raccoon population in Czechia was founded with individuals that either took a different invasion route or originated from an independent introduction event from its native range [33, 34]. Due to the random reduction of genetic diversity in comparison with the native range [31, 35, 36], those two clusters vary considerably both in neutral and functional genetic diversity [33, 34]. In contrast, the retention of functional MHC diversity in terms of supertype numbers and high individual allele divergence in the invasive range suggests selective pressure acting on the MHC-DRB locus [34]. The sharp differentiation found between the raccoon population from Czechia and those from Germany and Poland does not appear to be connected with habitat differentiation. In both regions, the raccoon inhabits wetlands and riparian habitat as well as urban areas that provide abundant food [37]. The raccoon is an opportunistic and omnivorous species and uses almost any available food resource in both types of habitats: rodents, birds, and their eggs, fish, and amphibians [37–39].

The studied population exhibited a high prevalence of helminth parasites, Digenea, and Cestoda [40]. Helminth infections trigger strong immune response, often establish chronic infections resulting in anaemia, malnutrition, growth impairment, cognitive deficiencies, immunopathology, and lowered fecundity [41–43]. Studied regions, inhabited by the two raccoon genetic clusters, do not appear to differ in the composition of parasite fauna found in native mammalian hosts. For example, *Isthiophora melis* (Digenea) that infected the studied raccoons is commonly found in native mammalian hosts (such as the fox *Vulpes vulpes*, raccoon dog *Nyctereutes procyonoides*, badger *Meles meles*, pine *Martes martes,* and stone marten *M. foina* or European mink *Mustela lutreola*) in both regions [40–42].

In the presented study, we aimed to evaluate the process shaping immune genetic diversity in invasive populations of raccoon that experienced a typical random reduction in genetic diversity during population establishment and spread. According to the theory, if genetic drift dominates selection, different MHC alleles will be randomly distributed across different populations. However, if pathogen-driven selection is strong enough, the frequency of those alleles will be shaped by distinct local parasite pressures. To test this, we studied the relationship between host genetic diversity in the functional MHC-DRB locus and the prevalence and infection intensity of raccoon intestinal parasites. We took advantage of the knowledge on two different genetic clusters present in the studied invasive raccoon populations that differ considerably in MHC-DRB allele frequencies [33, 34] to study allele-parasite relationships depending on the allele composition of each cluster. We also tested the temporal changes of those relationships. Our main goal was to reveal how host-parasite interactions influence MHC diversity depending on existing levels of variation. We expect that the MHC-DRB alleles present in each cluster as a result of genetic drift and population spread in the invasive range affect the level of intestinal parasite infection, which in turn should be essential for the success of a population in a new environment.

## Results

### MHC DRB locus diversity and population structure

**Fig. 1:**
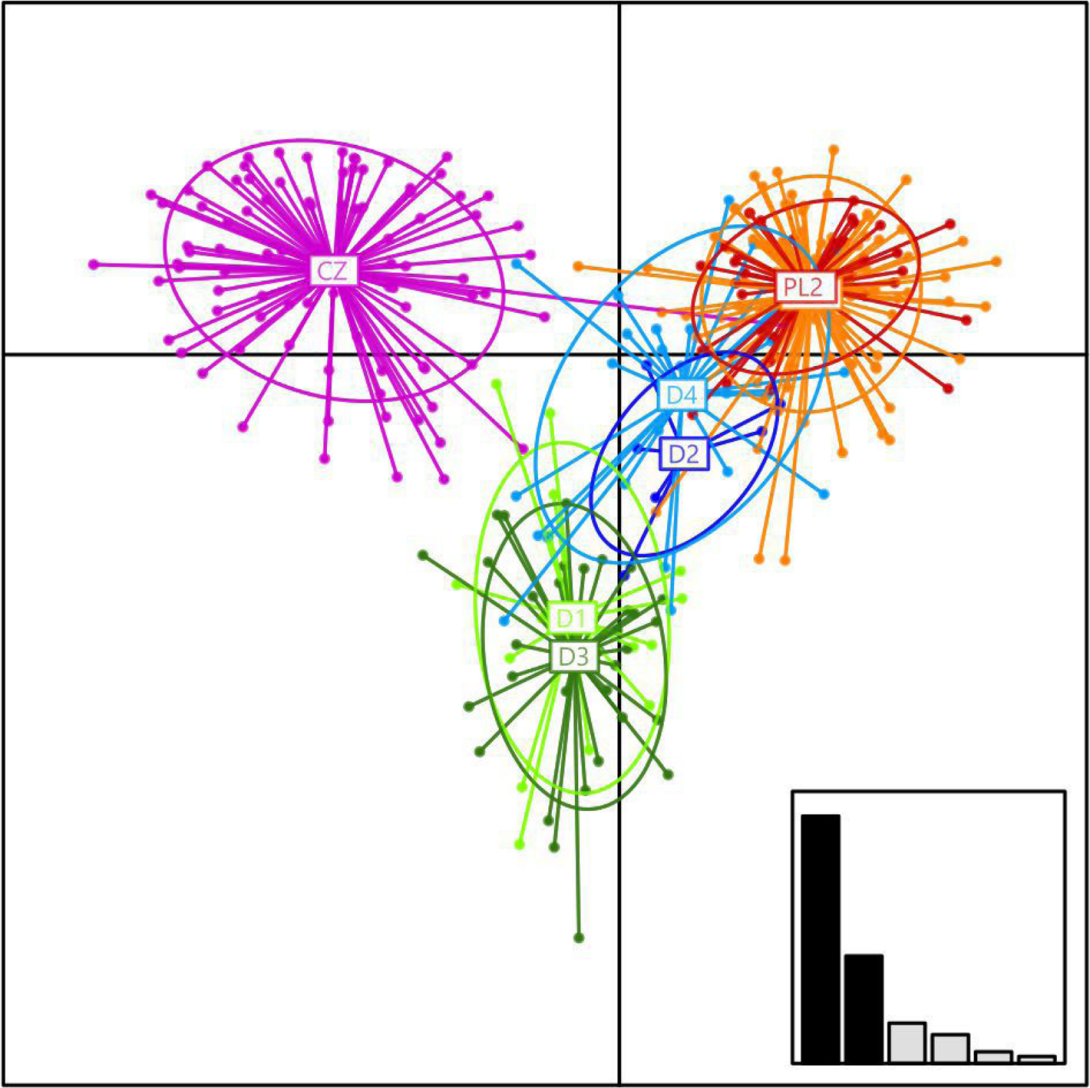
Scatterplot of the genetic differentiation across individuals resulting from discriminant analysis of principal components (DAPC) for the genetic structure of raccoon individuals MHC DRB locus. Individuals are presented as separate dots with colours denoting sampling locations and inclusion of 95% inertia ellipses. Abbreviations correspond to the sampling locations presented on Fig. 2. The inset shows the discriminant analysis (DA) eigenvalues

**Fig. 2:**
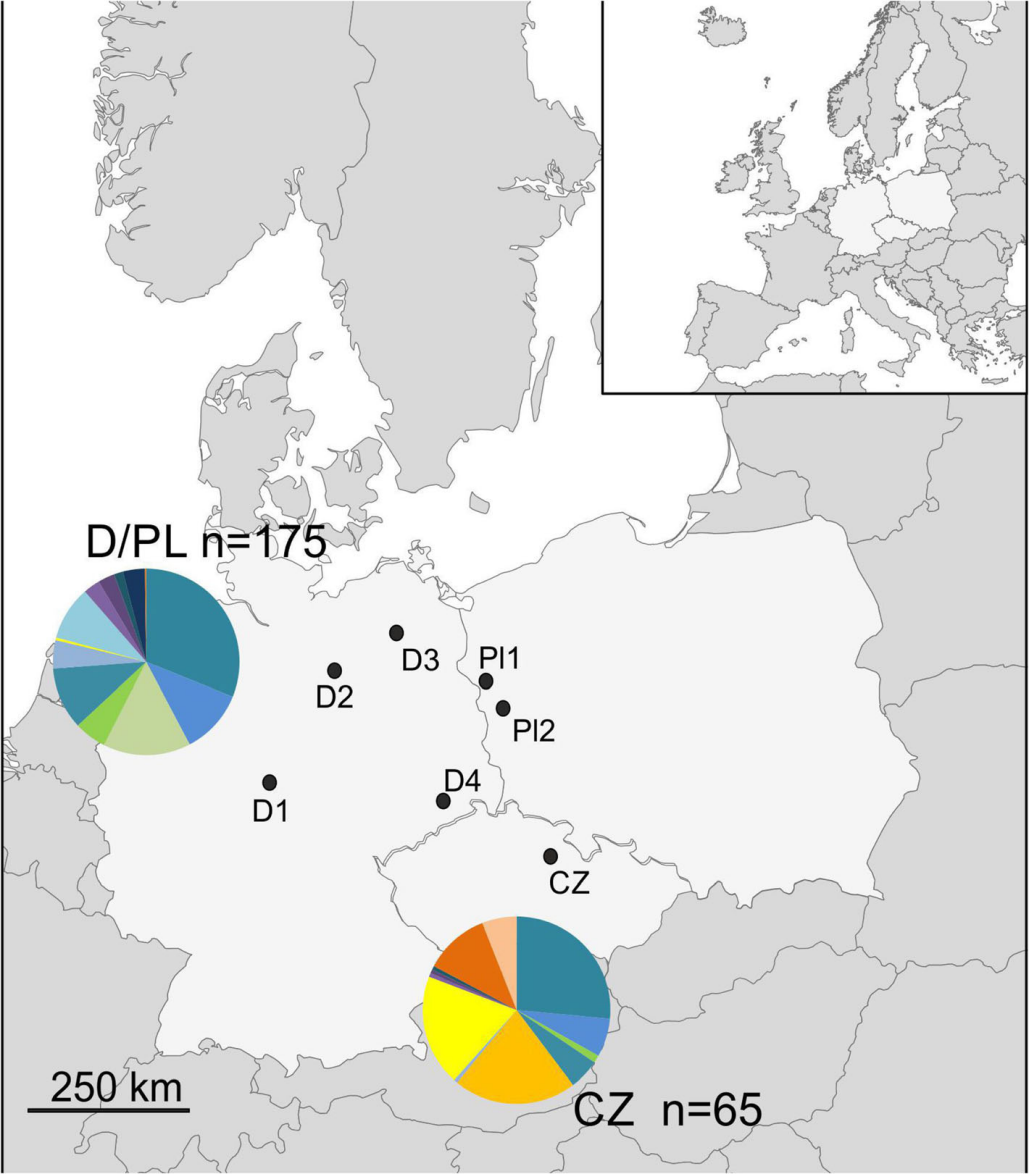
Geographical locations of invasive raccoon populations. MHC-DRB allele frequencies found in two genetic clusters shown by the pie charts. The dots represent the locations of sampled populations

Among the individuals used in this study, we detected 16 different MHC-DRB alleles. The number of alleles per individual ranged from 2 to 6. The mean individual amino acid diversity ranged from 0.197 to 2.788 for positively selected sites (AAdist_sel). The DAPC analysis performed for the MHC-DRB locus genotypes of individuals collected in four sampling sites in Germany, two in Poland and one in Czechia revealed clear differentiation between the Czech population and remaining sampling sites along the horizontal axis. No obvious differentiation was visible between individuals from Germany and Poland (Fig. 1). The distribution of alleles between sampling sites is shown in Fig. 2. In contrary, a strong subdivision between D/PL and CZ populations was also detected while analysing microsatellite loci in our previous study [34]. The differentiation between the CZ population and other invasive populations was comparable to the one between invasive European populations and native population from the USA.

### Parasite prevalence and intensity

A total of 237 raccoons (132 males and 105 females) were screened for parasite infection. The analyses revealed a total of 15 parasite taxa, representing Digenea, Cestoda, Nematoda, and Acanthocephala (Table 1). However, the presence of Acanthocephala representatives should be considered accidental, associated with the infection of a nonspecific host, being the parasites of fish and waterflow [44]. We detected pronounced differences in the basic parasitological indices between the D/PL and CZ sites. The prevalence of infection was significantly higher for raccoons from Germany and Poland than from Czechia when considering all parasite classes (0.69, CI = 0.61–0.75 vs 0.23, CI = 0.15–0.36), as well as for Digenea (0.54, CI = 0.47–0.61 vs 0.06 CI = 0.02–0.16) and Cestoda separately (0.20, CI = 0.14–0.27 vs 0.07, CI = 0.03–0.17, Fig. S1). Similarly, there was also a significantly higher abundance of all parasites, Digenea and Cestoda treated separately in the D/PL site in comparison to the CZ site (Fig. S2). In general, there was higher prevalence of all parasites in adult individuals and this difference was also significant for Cestoda prevalence and abundance. There was higher abundance of all parasites in females, but this result was not significant for the parasite classes treated separately (Tab. S1 and Fig. S1).

**Table 1:** The list of parasite taxa recorded in raccoons from Czechia (CZ) and Germany-Poland (combined, D/PL) with values of basic parasitological indices of infection

Parasite taxa	CZ (*n* = 62)			D/PL (*n* = 175)		
	Prevalence (%)	Infection intensity	Abundance	Prevalence (%)	Infection intensity	Abundance
*Isthmiophora melis*	4.8	70.3	3.4	50.3	101.1	50.8
Brachylaimidae sp_2	–	–	–	3.4	53.0	1.8
Brachylaimidae sp_1	–	–	–	0.6	51.0	0.3
*Plagiorchis* sp.	1.6	1.0	> 0.1	2.3	2.5	0.1
Digenea spp.	–	–	–	1.1	6.0	0.1
*Atriotaenia incisa*	–	–	–	12.0	41.4	4,9
*Mesocestoides* sp.	8.6	41.8	3.4	9.7	52.8	5.1
Cestoda spp.	–	–	–	0.6	1.0	> 0.1
*Baylisascaris procyonis*	–	–	–	4.6	9.5	0.4
Ancylostomatidae spp. (larvae)	4.8	8.3	0.4	4.6	3.4	0.2
*Toxascaris leonina*	–	–	–	0.6	1.0	> 0.1
*Aonchotheca putorii*	–	–	–	1.1	1.0	> 0.1
*Molineus patens*	–	–	–	0.6	2.0	> 0.1
*Polymorphus minutus*	6.5	53.3	3.4	–	–	–
*Acanthocephalus* sp.	1.6	1.0	> 0.1	1.1	1.5	> 0.1
Digenea (total)	6.5	53.0	3.4	54.3	97.8	53.1
Cestoda (total)	8.1	41.8	3.4	21.1	47.8	10.1

### Association between parasite infection and MHC alleles and diversity

**Fig. 3:**
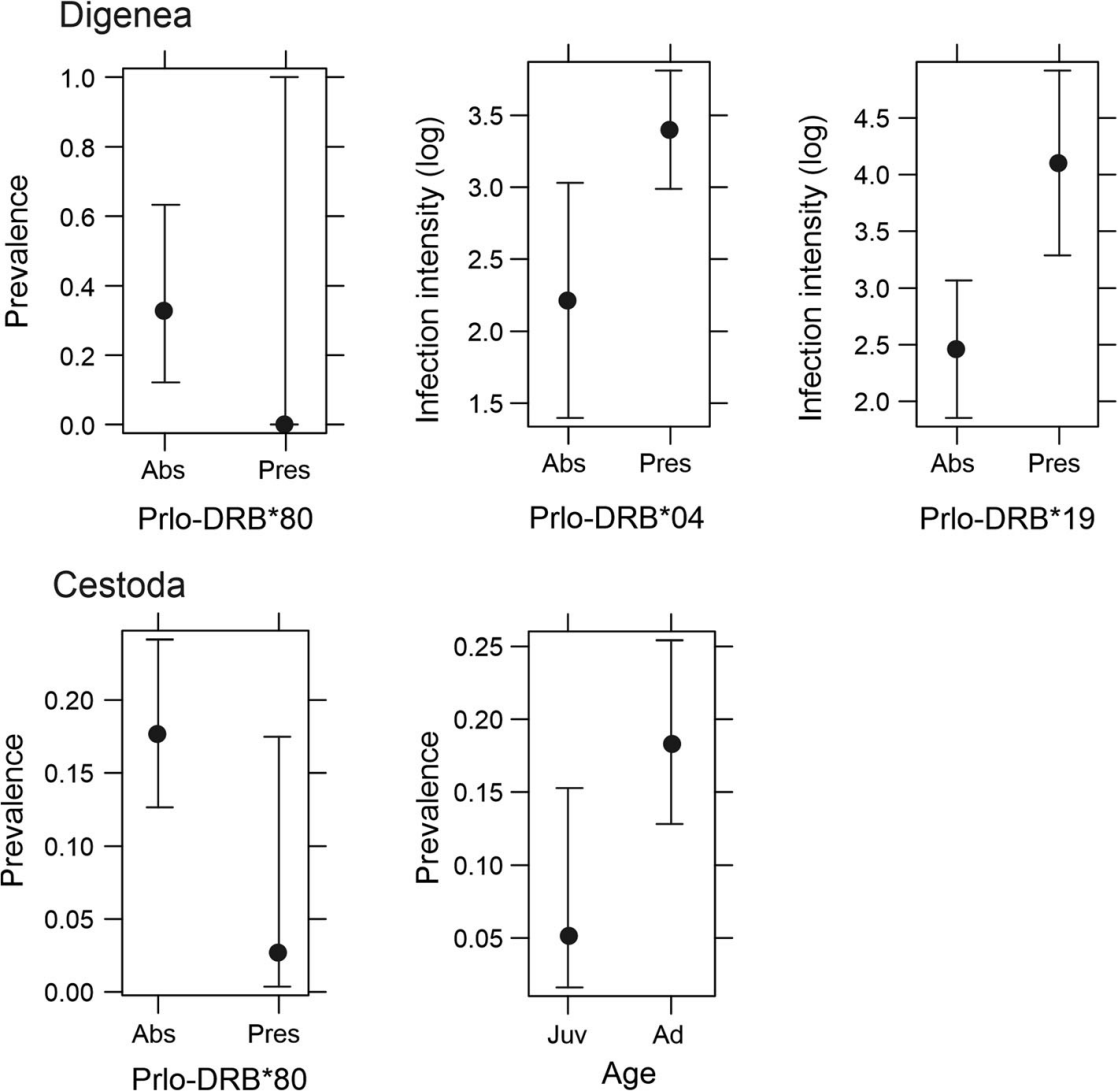
The association between prevalence or infection intensity of Digenea and Cestoda and presence/absence of particular alleles or host age estimated from the general and generalized linear mixed models. Only significant effects for MHC-DRB alleles are shown

Ten of 16 detected alleles had a frequency of over 10%. The presence/absence of two pairs of alleles was highly correlated (Prlo-DRB*04 and Prlo-DRB*14 as well as Prlo-DRB*16 and Prlo-DRB*62), therefore, we removed one allele from each pair from further analyses. Finally, we performed analyses of the association between parasite infection and allele presence/absence using 8 alleles (Table 2). We found associations between the Prlo-DRB*80 allele, present only in the Czech population, and Digenea prevalence (Table 2). In all raccoons possessing the Prlo-DRB*80 allele, no Digenea parasites were found Two alleles (Prlo-DRB*04 and Prlo-DRB*19) were associated with Digenea infection intensity (Table 2). The number of Digenea parasites was higher in raccoons with these alleles than in raccoons without them. Raccoons with the Prlo-DRB*04 allele were infected on average by 3.4 Digenea parasites (CI = 3.0–3.8), whereas raccoons without this allele by 2.2 parasites (CI = 1.3–3.1). Raccoons with the Prlo-DRB*19 allele were infected on average by 4.1 Digenea parasites (CI = 3.3–5.0), whereas raccoons without this allele by 2.4 (CI = 1.8–3.1; Fig. 3). The Prlo-DRB*19 allele was present only in raccoons from the German-Polish populations. No associations were found between the individual number of MHC-DRB alleles or individual allele divergence and parasite prevalence or intensity (Table 3).

**Table 2:** The results of a general and generalized linear model investigating the influence of different factors on the parasite prevalence or infection intensity of raccoons by gastrointestinal parasites from Digenea and Cestoda classes

Variables	DF	Prevalence		Infection intensity	
		LRT	*p*-value	LRT	*p*-value
Digenea					
Prlo-DRB*04	1	0.017	0.896	133.180	0.014*
Prlo-DRB*19	1	0.375	0.540	134.130	0.008**
Prlo-DRB*02	1	0.328	0.567	128.860	0.190
Prlo-DRB*69	1	1.319	0.251	128.020	0.349
Prlo-DRB*62	1	3.407	0.065.	127.200	0.802
Prlo-DRB*06	1	0.004	0.950	127.790	0.422
Prlo-DRB*102	1	0.287	0.592	128.280	0.285
Prlo-DRB*80	1	4.966	0.026*		
Sex	1	0.303	0.582	127.940	0.373
Age	1	2.221	0.136	128.380	0.266
Region	1	9.610	0.002**	127.460	0.574
Cestoda					
Prlo-DRB*04	1	0.189	0.664	65.433	0.591
Prlo-DRB*19	1	0.580	0.446	65.524	0.538
Prlo-DRB*02	1	0.042	0.838	65.338	0.659
Prlo-DRB*69	1	0.762	0.383	66.438	0.255
Prlo-DRB*62	1	0.920	0.338	65.154	0.920
Prlo-DRB*06	1	2.353	0.125	67.161	0.156
Prlo-DRB*102	1	0.039	0.843	65.964	0.365
Prlo-DRB*80	1	0.011	0.918	68.202	0.080.
Sex	1	0.533	0.465	65.909	0.382
Age	1	7.192	0.007**	65.817	0.412
Region	1	1.732	0.188	67.966	0.093.

* - p<0.05, ** - p<0.01, *** - p<0.001

*DF* degrees of freedom, *LRT* Likelihood Ratio Test

**Table 3:** The results of a general and genaralized linear model predicting the influence of different factors on the prevalence or infection intensity of Digenea and Cestoda parasites in raccoons

Variables	DF	Prevalence		Infection intensity	
		LRT	*p*-value	LRT	*p*-value
**Digenea**					
N alleles	1	0.646	0.422	134.000	0.104
AAdist_sel	1	0.012	0.913	131.380	0.873
Sex	1	0.005	0.943	131.590	0.630
Age	1	1.878	0.171	132.210	0.356
Region		49.262	0.000***	131.530	0.682
**Cestoda**					
N alleles	1	0.163	0.686	55.953	0.375
AAdist_sel	1	1.308	0.253	57.394	0.135
Sex	1	0.201	0.654	55.949	0.376
Age	1	6.671	0.010**	55.699	0.465
Region		5.226	0.022*	55.231	0.796

* - p<0.05, ** - p<0.01, *** - p<0.001

*DF* degrees of freedom, *LRT* Likelihood Ratio Test. AAdist_sel - the mean individual amino acid divergence for sites found to be under positive selection between alleles born by an individual

**Fig. 4:**
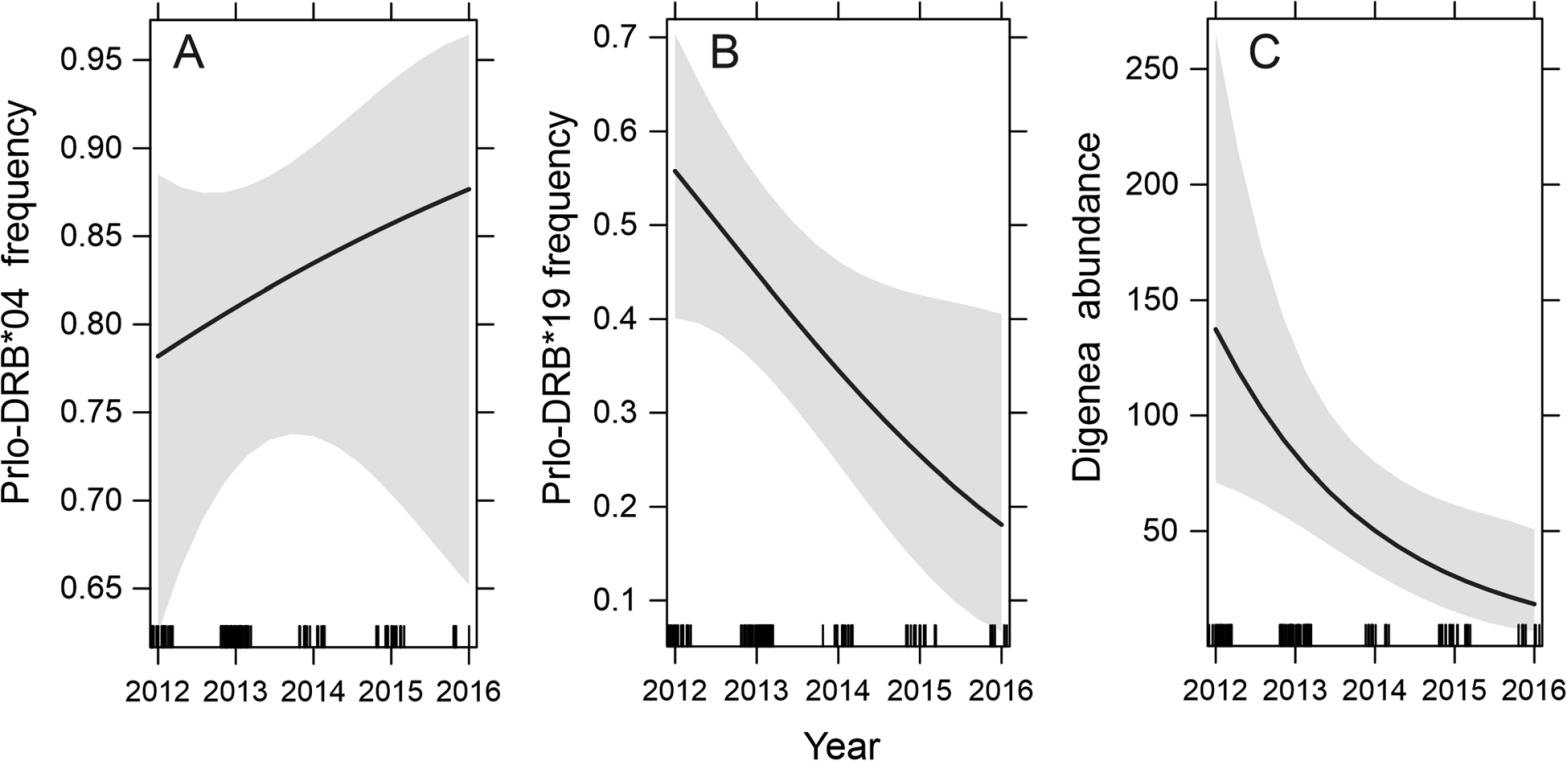
The overtime changes in the frequency of the Prlo-DRB*04 allele (A) Prlo-DRB*19 allele (B) and the abundance of Digenea parasites (C) in raccoons from Warta Mouth National Park (PL1 and PL2 see Fig. 2). The effects of raccoon sex and age are not shown

The frequency of the Prlo-DRB*04 allele did not change significantly during the 5 years of study in the Warta Mouth National Park. In contrast, the frequency of the Prlo-DRB*19 allele significantly decreased (Table 4, Fig. 4). In 2012, the frequency of Prlo-DRB*19 was 0.56 (CI = 0.40–0.70) and decreased to 0.18, (CI = 0.07–0.41) in 2016. There was no significant variation in the frequency of any other alleles over time (data not presented). Digenea abundance decreased from an average of 137.4 (CI = 71.1–265.3) in 2012 to 18.3 (CI = 6.6–50.7) in 2016 (Table 4, Fig. 4).

**Table 4:** Parameter estimates and test statistics from the models explaining the overtime changes in the frequency of the Prlo-DRB*04 and Prlo-DRB*19 alleles and the abundance of Digenea parasites in raccoons from Warta Mouth National Park

Variables	Estimate	SE	z value	*p*-value
Prlo-DRB*04				
Intercept	−343.208	468.679	−0.732	0.464
Year	0.171	0.233	0.735	0.462
Sex (M)	−0.2418	0.513	−0.471	0.638
Age (juv)	0.3081	0.7207	0.427	0.669
Prlo-DRB*19				
Intercept	875.8694	398.1068	2.200	0.028*
Year	−0.4354	0.1978	−2.201	0.028*
Sex (M)	0.5999	0.4199	1.429	0.153
Age (juv)	0.1312	0.5468	0.240	0.810
**Digenea abundance**				
Intercept	1018.3888	366.4658	2.779	0.0055**
Year	−0.5038	0.1820	−2.767	0.0057**
Sex (M)	0.3499	0.4243	0.825	0.4095
Age (juv)	−0.1653	0.5450	−0.303	0.7617

* - p<0.05, ** - p<0.01 *** - p<0.001

## Discussion

In the study presented, we used a large dataset spanning 5 years and including raccoon populations most probably originating from different introduction events. As a result, studied populations present strong population structure, a consequence of not only different MHC-DRB allele frequencies but also different sets of MHC-DRB alleles present in both sites. We provided an insight into how selection, imposed by intestinal parasites, shapes MHC class II diversity after a population bottleneck and how this diversity is shaped over a temporal scale. The ability of populations to adapt to local conditions in spatially heterogeneous environments depends on the level of genetic variation. In invasive populations, this adaptability is strongly affected by a stochastic reduction in genetic diversity, due to founder events and population bottlenecks. Our results suggest that despite a stochastic loss of functional immune diversity, invasive populations are able to respond quickly to environmental factors, such as local parasite pressure. The differences in the MHC-DRB allele composition are most probably the result of demographic processes that took place during population establishment. Still, the different MHC-parasite associations found in both clusters suggest ongoing selection imposed on the invasive raccoon populations by local parasites. These arising relationships are strongly related to the level of genetic diversity created previously by a neutral process.

In raccoon populations from Germany and Poland, we found MHC-parasite associations that were related to alleles present only in these sites. We found that possession of the Prlo-DRB*19 allele was related to a higher probability of Digenea infection. The positive association between Prlo-DRB*19 and infection intensity suggests that this variant acts as a susceptibility allele, a kind of relationship often reported in earlier studies [23, 25, 45–47]. As expected, we detected a significant decrease in the frequency of the Prlo-DRB*19 allele over sampling years (2012–2016, Table 4, Fig. 4). Importantly, this process was observed despite the increase in raccoon population census numbers and population density, both in Germany and Poland during the time of the study [32], which is in line with the pattern of frequency-dependent selection imposed by parasites on MHC-DRB alleles within this region. The drop in the frequency of this susceptibility allele is pronounced, taking into account only five years long sampling period. Nevertheless, in multi-allele MHC loci, such rapid changes are the effect of not only increased mortality of susceptibility allele bearers caused by parasites. In such loci, the effect of overdominant selection, the high level of polymorphism, and a high rate of gene flow due to copy number variation act synergistically and impose an order of magnitude higher changes in allele frequencies in comparison with neutral loci [48]. The presence of the susceptibility allele in the D/PL genetic cluster is reflected in the prevalence of Digenea parasites that was two times higher than in the Czech population, where this allele was absent. We believe that this difference is not due to environmental differences affecting the presence of Digenea parasites. The low prevalence of Digenea in Czech raccoons (6.5%) versus 54.3% in Polish and German raccoons is in contradiction to its frequent occurrence in other mammalian hosts. Digenea parasites are common in red fox, raccoon dog, badger, pine and stone marten or European mink [49–51] and intermediate hosts in the region where the Czech population was sampled [52–54]. The differences in infection levels also cannot be explained by the diet preferences of raccoons from Czechia in comparison to raccoons from the D/PL region, as both genetic clusters inhabit wetland regions with a high abundance of intermediate snail hosts [37, 52–54]. The second genetic factor that seems to influence the level of Digenea infection in the Czech population is the Prlo-DRB*80 allele, which is found only there and no Digenea infection was found in individuals bearing this allele. Although the absence of a susceptibility allele and the presence of a resistance allele were found to affect the level of Digenea infection in the Czech population, we cannot conclude which factor plays a more important role in maintaining the Digenea infection at the low level. Digenea parasites also invade raccoons in their native range [28, 55], therefore there is a possibility that these associations are not novel and developed before the raccoon invasion in Europe. In this light, the accidental loss of those protection alleles during the establishment of the raccoon in the German-Polish region, which are pooled in one genetic cluster, resulted in significantly higher levels of Digenea parasitic infections. This finding suggests the role of standing genetic variation in coping with parasite pressure occurring in a novel environment.

The positive association between the MHC-DRB allele and the intensity of Digenea infection was also found for the Prlo-DRB*04 allele that exhibited high frequencies (0.8–0.9, apart from the D1 site where the frequency is 0.44) through whole studied invasive raccoon range. Although the association suggests that the individuals bearing the allele are more susceptible to infection, we did not detect a significant change in allele frequency over time. Taking into account that we observed the decrease of another MHC-DRB variant that is acting as susceptibility allele for Digenea infection, it can be expected that such frequency change should also be observed for the Prlo-DRB*04. Nevertheless, in this case, we suspect that high and constant frequency of this allele across invasive raccoon populations may be rather connected to a different factor. Viral infections that cause high mortality rates, like rabies and distemper, play a predominant role in raccoon population dynamics in the native range [45, 56] and impose intense selective pressure. In the study of Srithayakumar et al. [46] and Kyle et al. [36], the Prlo-DRB*04 allele was proved to give resistance to rabies in the native raccoon range. What is more, the variability in raccoon immune response to rabies and survival, which may be related to this specific allele, were also detected [47, 57]. In the introduced raccoon range, rabies was observed rarely. In Europe, 142 cases were noted between 1990 and 2010, most of them outside of our study region in Ukraine, [28]. Therefore, the very high frequencies of Prlo-DRB*04 allele we found in this study over the whole study period are rather the result of intense selective pressure that took place before the raccoon introduction to Europe and the factor that gives raccoons protection to rabies in the invasive range.

The lack of associations between MHC genetic diversity (the number of MHC-DRB alleles) and parasite prevalence and infection intensity on the population level in our study seems to be consistent with a recent loss of genetic diversity through a population bottleneck. Many MHC-DRB alleles were lost in comparison with the native population [34]. Even if an association between the level of MHC-DRB diversity and the level of infection exists in the native raccoon range, it would be lost during genetic bottleneck when invasive population was established. Additionally, the association between MHC heterozygosity (expressed by the number of alleles, or similar rate) should be expected when the host is infected by multiple parasites that select for persistence of numerous alleles [58], while here we detected two predominant parasites, Digenea and Cestoda, and we should rather expect allele-specific associations. Similarly, we did not find any evidence for the adaptive advantage of mean individual aminoacid divergence at MHC-DRB locus, although according to theory, possessing differentiated alleles, that can give protection from a wider range of pathogens should be connected to lower infection levels [59]. In our study, the overall prevalence of different classes of intestinal parasites was rather low. Taking into account that allele divergence substantially increases the range of the potential antigen repertoire, in the environment with relatively low parasite enemies, it may be disadvantageous to maintain highly divergent allele combinations.

## Conclusions

Overall, our findings suggest that intestinal parasites can exert selective pressure on the invasive hosts with lowered levels of immune genetic diversity and reduced genetic variation and contribute to promoting local adaptation over time. Numerous studies on MHC variation in natural populations report on the role of genetic drift in shaping MHC variation. The most important result of our study is consistent with the pattern of random genetic drift, that created two different genetic clusters in invasive raccoon range, what in turn imposed completely different allele-parasite associations what seems to affect the prevalence of parasites in populations from both genetic clusters. Our results also suggest that despite the reduced level of immune genetic diversity the frequency-dependent selection in response to pathogen pressure could be still observed in the invasive population. Overall, these findings underline the role of standing genetic variation in shaping host-parasite relationships and gives empirical support that may explain the different success of invasive populations.

## Methods

### Sample collection

We collected 237 raccoon carcasses from the European part of the introduced range of the species: 112 from Poland, sites PL1 and PL2, 63 from Germany, sites D1-D4, and 62 from one site in Czechia (CZ) between 2012 and 2017. The localities of the sampling sites are shown in Fig. 2. The carcasses were obtained from hunters culling raccoons as part of game management activities or raccoons killed by car accidents collected along roads. We collected only animals that were proven to be killed several hours earlier, with no signs of decomposition. The tissue samples for DNA analyses were collected as dried ear fragments or ethanol-preserved tissue. The carcasses were kept frozen at -20 °C prior to dissection.

### DNA isolation, MHC-DRB locus amplification and genotyping

The genetic data on MHC-DRB diversity was obtained as a result of the previous study [34]. Here we give brief details of laboratory procedure and genotyping protocol. DNA was extracted from dried ear fragments or ethanol-preserved tissue using the NucleoSpin Tissue Kit (Macherey and Nagel, Dueren, Germany) according to the manufacturer’s protocol. To assess MHC diversity, we focused on the MHC-DRB locus, as it is the most polymorphic MHC class II loci. The diversity of the 184 bp fragment of exon 2 that contains the functionally important protein binding region (PBR) was assessed by amplicon sequencing using the Illumina Miseq platform. The sequencing primers consisted of DRB 5c or DRB 3c primers followed by a unique 6-bp barcode and Illumina-specific primers. Amplification was performed with HotStar Master Mix (QIAGEN), and the reaction was run for 27 cycles at 95 °C for 30 s, 66 °C for 30 s, 72 °C for 1 min 30 s. We used a combination of 12 forward and 8 reverse uniquely barcoded primers in 96-well PCR plates. We included one negative control per 16 samples, and 24 samples were run as duplicates to control for sequencing errors. The read merging, filtering, quality control and preliminary control of length, coverage and frequency of the most abundant variants, as well as the final genotyping of the MHC-DRB locus, was performed using the AmpliSAS pipeline [60]. The detailed description of Illumina Miseq sequencing and genotyping procedure is given in [34].

### MHC-DRB diversity and population structure

To test if the population structure at the MHC-DRB locus of the raccoons used in this study, collected over a large part of its invasive range, follows the previously described pattern, we used the discriminant analysis of principal components (DAPC) implemented in the R package adegenet [61]. Multivariate analyses are the method of choice when the assumptions of Hardy-Weinberg and linkage equilibrium within populations are violated as they can summarize the genetic variability without making strong assumptions about an evolution model [61]. DAPC uses principal component analysis (PCA) to reduce the number of variables used in the discriminant analysis (DA) to only use the variables that are uncorrelated. This approach maximizes between-group differences while minimizing within-group variance, thus enabling DAPC to detect subtle differences between populations. First, using the function *find.clusters*, we identified the number of clusters that best reflects the genetic structuring in the data without a prior assignment of samples to given populations, using BIC scores [Bayesian Information Criterion, [61]]. A cross-validation function *(Xval*.*Dapc*) was used to select the optimal number of principal components to be retained. Next, we used DAPC to generate assignment probabilities to actual populations (sampled sites). Spatial relationships between populations were visualized on the scatterplots. The associations between MHC-DRB diversity and parasite infection were performed using population grouping obtained in DAPC analysis.

The level of MHC-DRB diversity characterizing each identified genetic cluster was expressed as the individual number of alleles. The MHC-DRB alleles present in raccoons were also grouped into functional supertypes [34]. MHC alleles of the same supertype encode biochemically similar amino acids at antigen-binding sites and thus the molecules bind similar antigens. Therefore, the alleles of different supertypes should have different functional values [62, 63]. Nevertheless, in this study, we performed the analysis using MHC-DRB alleles. As our study system comprised individuals from highly bottlenecked populations characterized by a limited number of MHC-DRB alleles, searching for associations between specific alleles and parasite infection enabled us to retain a higher resolution of our analysis. To assess the level of dissimilarity of the alleles borne by an individual, we estimated the mean individual amino acid divergence for sites found to be under positive selection (AAdist_sel) using MEGA7 [64]. Positively selected sites were detected using phylogenetically controlled models of selection: MEME, FUBAR, SLAC, and FEL [65] as described in [34].

### Parasite screening

Each animal was sexed, weighed and body length was measured during dissection. Based on body length, weight and month of death, animals were classified as juvenile or adult. The whole alimentary tract was examined and macroscopically screened for the presence of helminths. Next, the gut contents were rinsed in 0.9% physiological salt, decanted and examined under a stereomicroscope. The internal organs: trachea, lungs, heart, liver, gall bladder, spleen, kidneys and urinary bladder were separated and examined with standard helminthological techniques. All the isolated helminths were rinsed, counted and preserved in 70 or 90% ethyl alcohol. The fixed digeneans and tapeworms were stained with borax carmine and contrasted with acidified ethyl alcohol. The helminths were dehydrated in a graded alcohol series and, following clearing in clove oil, mounted on microscope slides with Canada balsam. In the case of hookworms, the procedure was the same except that the staining was skipped. The small nematodes were cleared in glycerol, while large specimens were identified based on morphological characteristics.

As our goal was to assess MHC-parasite relationships in populations showing a pronounced inter-population structure and probably established from two different introduction events (Biedrzycka et al. 2014, 2019), we combined the data into two regions: (1) Czechia and (2) Germany-Poland. To analyse the differentiation between both regions in terms of the prevalence of each parasite class (Digenea, Cestoda, Nematoda, and Acanthocephala) as well as all parasites pooled, we used a generalized linear model with binomial error distribution. We added to the models: (1) site, (2) host sex and (3) host age (as we expect that both host sex and age may affect the level of parasite infection) as explanatory variables. To analyse the differences between regions in parasite abundance (mean number of parasites per raccoon, including uninfected raccoons [66], a generalized linear model with negative binomial error distribution was used with site, host sex, and age as explanatory variables. The models were computed in the ‘MASS’ package in R v. 3.4.2 [67].

### Statistical analyses

Due to low overall prevalence, in order to achieve desirable statistical power, in the models of MHC-infection relationships we considered infections with *Digenea* and *Cestoda* rather than with particular parasite species. Such an approach seems justified biologically, as the host immune system is limited in its ability to distinguish between different classes of helminth species, which leads to many-to-many gene-parasite associations [68, 69]. This approach is also widely used in the studies of MHC-parasite associations (eg [42, 43, 49].). Nematoda and Acanthocephala were excluded from the analysis of the association between MHC-DRB and parasite infection due to low prevalence. In the models testing the effect of the presence of specific MHC-DRB alleles on parasite infection, we included alleles with a frequency higher than 10%. Prior to the analyses, we tested for the presence of an autocorrelation between the presence/absence of any allele pairs. If such a correlation was detected, we randomly removed one of the alleles from the pair from further analyses.

We tested the genetic effects on both the qualitative (parasite prevalence) and quantitative characteristics of parasite infection (infection intensity: number of parasites per individual, excluding uninfected individuals [66];). Infection intensity was log-transformed prior to analysis. Our goal was first to analyse the association between specific MHC-DRB alleles and parasite infection, and second, to check for the effect of possessing more diverse sets of MHC alleles on parasite infection. Therefore, we performed two separate analyses: (1) on the association between the presence/absence of particular MHC alleles and susceptibility, and (2) the influence of overall MHC diversity in an individual and susceptibility. As the measure of the MHC diversity for each individual, we used the number of MHC-DRB alleles. To assess the role of individual differentiation of alleles, we used the mean individual amino acid allele divergence for sites found to be under positive selection (AAdist_sel). In total, we applied four models: two explaining parasite prevalence in relation to the presence/absence of particular MHC alleles or overall MHC diversity and two for infection intensity in relation to the presence/absence of particular MHC alleles or overall MHC diversity. In these two sets of models, individual parasite prevalence or intensity was the dependent variable and the presence/absence of 8 alleles or MHC diversity (3 measures described above) were included as the fixed variables. Besides, raccoon sex, age (subadult and adult), and region (Czechia and Germany/Poland) were included in all models as the fixed variables (as they can be important predictors for parasite infection). We tested the relation between parasite infection and MHC-DRB diversity using generalized (for parasite prevalence; GLM with binomial error distribution) and general linear models (for infection intensity; LM). GLM and LM were calculated with glm and lm functions respectively, applied in the LME4 package in R v 3.4.2 [67]. To assess whether a given variable significantly explains the variance, we compared the models with and without the variable using a likelihood ratio test.

We also analysed changes in parasite infection levels and the frequency of alleles potentially associated with the parasite class (*Digenea* and *Cestoda*) over 5 years of the study period. This analysis was performed only for sites PL1 and PL2 from which we had a large sample size over all consecutive 5 years (2012–2016). As the parasite infection intensity is calculated only for infected individuals (subset of the samples), to compare the variation in the frequency of potential alleles associated with the parasite group and parasite infection levels in consecutive years, we pooled the parasite prevalence and intensity in one index – parasite abundance (number of parasites per individual, including uninfected individuals [66];) to use the same number of samples in both analyses. Models for parasite abundance were fitted using a generalised linear model with negative binomial error distributions and a log-link function (glm.nb function) implemented in the MASS package [70]. The variation in the frequency of MHC-DRB alleles associated with a parasite class over time was analysed using generalized linear models with binomial error distribution, including year, raccoon sex and age as response variables. All analyses were performed in R statistic software v 3.2.3 [67].

## Supplementary information

**Additional file 1: Table S1.** The results of a generalized linear model predicting the influence of population, sex and age factors on the prevalence and abundance of all intestinal parasites and detected parasite classes.

**Additional file 2: Fig. S1.** The effects of population, sex and age on prevalence of all intestinal parasites and different parasite classes predicted by a generalized linear model.

**Additional file 3: Fig. S2.** The effects of population, sex and age on the abundance of all intestinal parasites and different parasite classes predicted by a generalized linear model.

## Data Availability

The results of individual genotyping of MHC-DRB locus obtained as a result of genotyping procedure of Illumina sequencing files and scanning for intestinal parasites will be deposited in Open Science Framework Repository service upon publication.

## Abbreviations

MHC: Major histocompatibility complex
CZ: Population from Czechia
PL1, PL2: Populations from Poland
D1, D2, D3, D4: Populations from Germany
PBR: Protein binding region
DAPC: Discriminant analysis of principal components
PCA: Principal component analysis
DA: Discriminant analysis
BIC: Bayesian Information Criterion
AAdist_sel: Amino-acid divergence for sites found to be under positive selection
GLM: Generalized linear model
LM: General linear models
