# Supplementary material for: Host-parasite interactions in non-native invasive species are dependent on the levels of standing genetic variation at the immune locus
Source: BMC Evol Biol. 2020 Apr 16;20:43. doi: 10.1186/s12862-020-01610-x (PMC7164242; doi:10.1186/s12862-020-01610-x)
Supplement: Supplementary file 1 — Additional file 1: Table S1. The results of a generalized linear model predicting the influence of population, sex and age factors on the prevalence and abundance of all intestinal parasites and detected parasite classes. [file 12862_2020_1610_MOESM1_ESM.docx]

Table S1. The results of a generalized linear model predicting the influence of population, sex and age factors on the prevalence and abundance of all intestinal parasites and detected parasite classes.

| Variables | Estimate | SE | Z value | P |  |
| --- | --- | --- | --- | --- | --- |
| **Prevalence** | | | | | |
| **All parasites** | | | | | |
| Intercept | -1.0811 | 0.3465 | -3.120 | 0.00181 | ** |
| Population (D/PL) | 1.9477 | 0.3425 | 5.686 | 1.3e-08 | *** |
| Sex (M) | 0.1558 | 0.2902 | 0.537 | 0.59129 |  |
| Age (juv) | -0.7791 | 0.3510 | -2.219 | 0.02645 | * |
| **Digenea** |  |  |  |  |  |
| Intercept | -2.5680 | 0.5461 | -4.703 | 2.57e-06 | *** |
| Population (D/PL) | 2.8525 | 0.5398 | 5.284 | 1.26e-07 | *** |
| Sex (M) | -0.0145 | 0.2948 | -0.049 | 0.961 |  |
| Age (juv) | -0.5192 | 0.3686 | -1.408 | 0.159 |  |
| **Cestoda** |  |  |  |  |  |
| Intercept | -2.3335 | 0.5139 | -4.541 | 5.61e-06 | *** |
| Population (D/PL) | 1.1091 | 0.5055 | 2.194 | 0.0282 | * |
| Sex (M) | 0.1751 | 0.3541 | 0.495 | 0.6209 |  |
| Age (juv) | -1.3776 | 0.6258 | -2.202 | 0.0277 | * |
| **Nematoda** |  |  |  |  |  |
| Intercept | -2.8447 | 0.6499 | -4.377 | 1.2e-05 | *** |
| Population (D/PL) | 0.8141 | 0.6427 | 1.267 | 0.205 |  |
| Sex (M) | -0.3476 | 0.4600 | -0.756 | 0.450 |  |
| Age (juv) | 0.2092 | 0.5431 | 0.385 | 0.700 |  |
| **Acanthocephala** | | | | | |
| Intercept | -2.5372 | 0.6837 | -3.711 | 0.000206 | *** |
| Population (D/PL) | -2.0174 | 0.8513 | -2.370 | 0.017793 | * |
| Sex (M) | 0.0098 | 0.7928 | 0.012 | 0.990177 |  |
| Age (juv) | 0.3838 | 0.8740 | 0.439 | 0.660541 |  |
| **Abundance** | | | | | |
| **All Parasites** | | | | | |
| Intercept | 2.6326 | 0.3754 | 7.012 | 2.34e-12 | *** |
| Population (D/PL) | 2.0101 | 0.3672 | 5.475 | 4.38e-08 | *** |
| Sex (M) | -0.6487 | 0.3232 | -2.007 | 0.0448 | * |
| Age (juv) | -0.5752 | 0.3975 | -1.447 | 0.1479 |  |
| **Digenea** |  |  |  |  |  |
| Intercept | 0.6983 | 0.4807 | 1.453 | 0.146 |  |
| Population (D/PL) | 3.4121 | 0.4689 | 7.276 | 3.44e-13 | *** |
| Sex (M) | -0.3206 | 0.4095 | -0.783 | 0.434 |  |
| Age (juv) | 0.7815 | 0.5016 | 1.558 | 0.119 |  |
| **Cestoda** |  |  |  |  |  |
| Intercept | 1.4554 | 0.7778 | 1.871 | 0.0613 | . |
| Population (D/PL) | 1.9274 | 0.7652 | 2.519 | 0.0118 | * |
| Sex (M) | -1.0816 | 0.6702 | -1.614 | 0.1065 |  |
| Age (juv) | -1.9696 | 0.8339 | -2.362 | 0.0182 | * |
| **Nematoda** |  |  |  |  |  |
| Intercept | -0.8595 | 0.8758 | -0.981 | 0.326 |  |
| Population (D/PL) | 0.5481 | 0.8568 | 0.640 | 0.522 |  |
| Sex (M) | -0.2514 | 0.7507 | -0.335 | 0.738 |  |
| Age (juv) | -0.1039 | 0.9239 | -0.112 | 0.910 |  |
