# Supplementary figures and images for: Host-parasite interactions in non-native invasive species are dependent on the levels of standing genetic variation at the immune locus
Source: BMC Evol Biol. 2020 Apr 16;20:43. doi: 10.1186/s12862-020-01610-x (PMC7164242; doi:10.1186/s12862-020-01610-x)

# All parasites

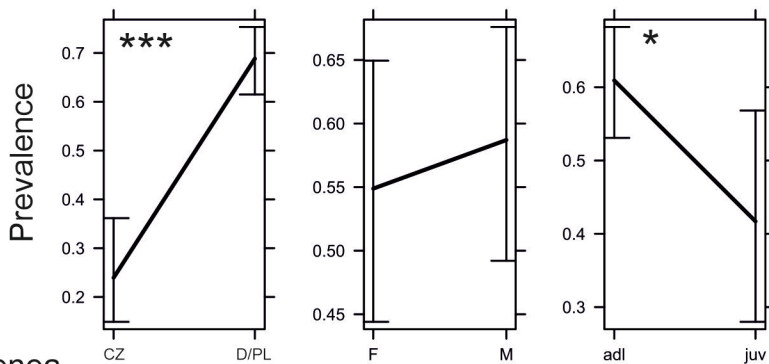

## Digenea

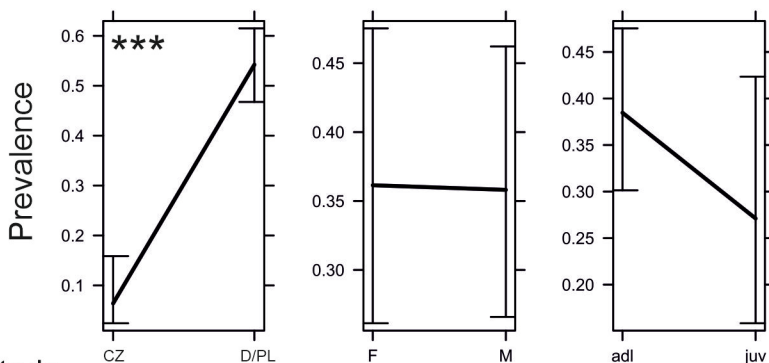

## Cestoda

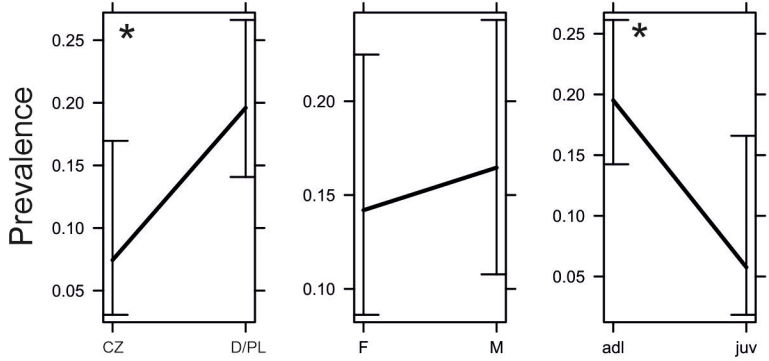

## Nematoda

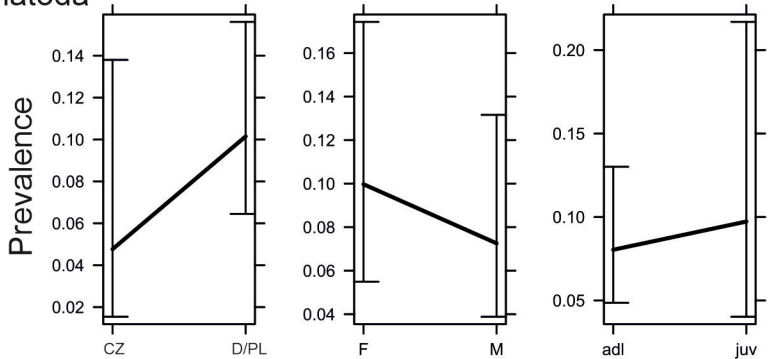

## Acanthocephala

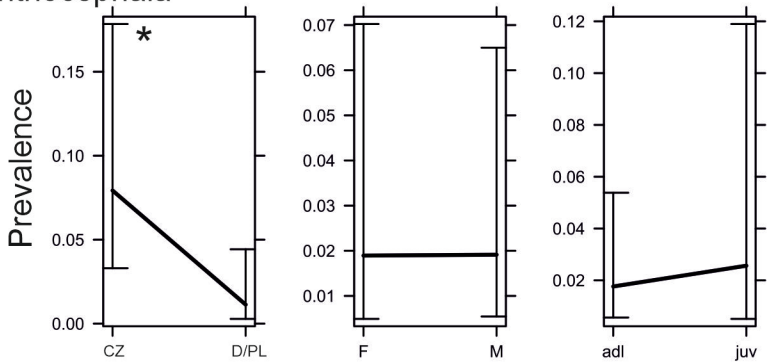

Population

Sex

Age

Supplement: Supplementary file 2 — Additional file 2: Fig. S1. The effects of population, sex and age on prevalence of all intestinal parasites and different parasite classes predicted by a generalized linear model. [file 12862_2020_1610_MOESM2_ESM.pdf]

# All parasites

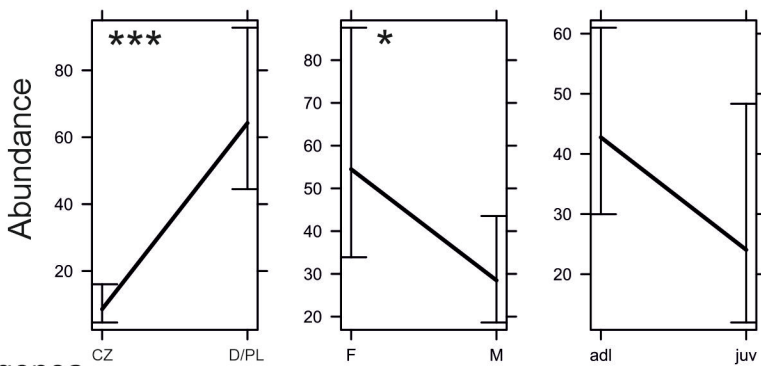

## Digenea

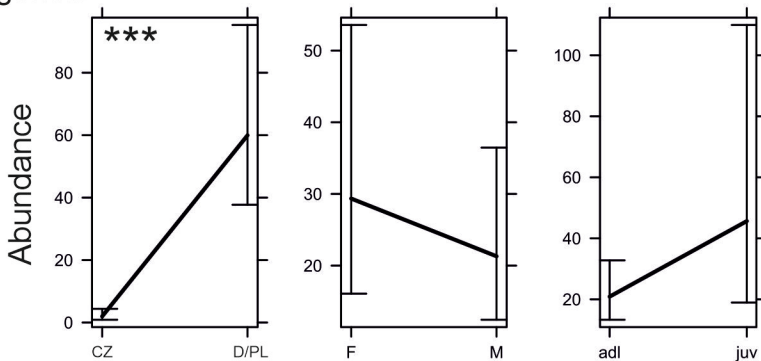

## Cestoda

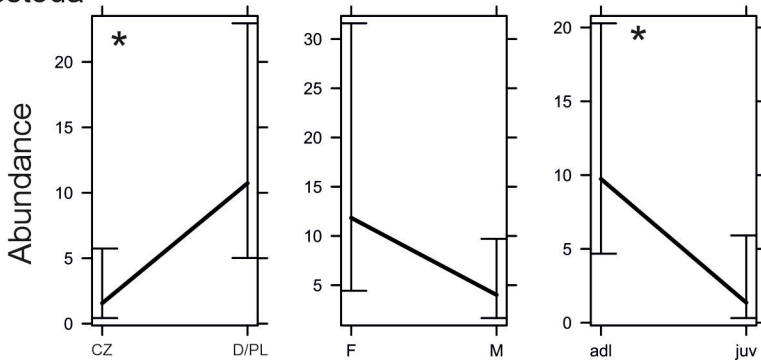

## Nematoda

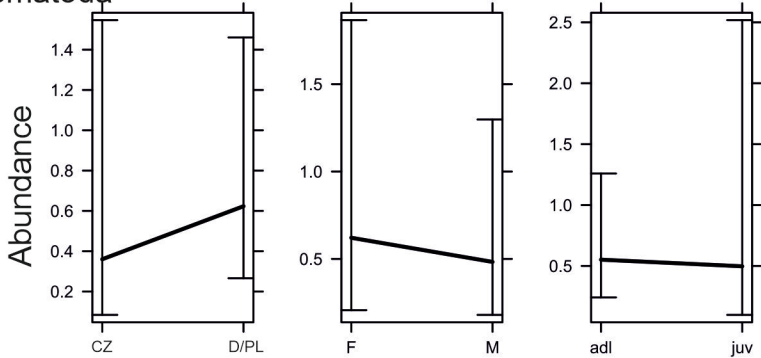

Population

Sex

Age

Supplement: Supplementary file 3 — Additional file 3: Fig. S2. The effects of population, sex and age on the abundance of all intestinal parasites and different parasite classes predicted by a generalized linear model. [file 12862_2020_1610_MOESM3_ESM.pdf]
